# Upcycling of SARS-CoV-2 Rapid Antigen Test Cassettes into Flame Retardant Plastics

**DOI:** 10.3390/ma17102384

**Published:** 2024-05-16

**Authors:** Tadej Slatinek, Janez Slapnik

**Affiliations:** Faculty of Polymer Technology, Ozare 19, 2380 Slovenj Gradec, Slovenia; tadej.slatinek@ftpo.eu

**Keywords:** SARS-CoV-2, COVID-19, medical waste, rapid antigen tests, lateral flow immunoassay, mechanical recycling, upcycling, high-impact polystyrene, phosphorous-based flame retardants

## Abstract

The COVID-19 pandemic resulted in the generation of large quantities of medical waste and highlighted the importance of efficient waste management systems. One good example of this is rapid antigen tests, which contain valuable resources, and which are usually incinerated after their use. The present study aimed to evaluate the potential of waste rapid antigen test cassettes (RATCs) as a resource for the preparation of sustainable flame-retardant plastics. Milled RATCs were compounded with different concentrations (10–30 wt.%) of aluminium diethylphosphinate (ADP) and injection moulded into test specimens. Prepared samples were exposed to ultraviolet (UV) ageing for varying durations and characterised by Fourier-transform infrared spectroscopy (FT-IR), differential scanning calorimetry (DSC), thermogravimetric analysis (TGA), dynamic mechanical analysis (DMA), tensile tests, Charpy impact tests, and vertical burning tests. FT-IR analysis revealed that RATCs are composed mainly of high-impact polystyrene (HIPS), which was further confirmed by suitable glass transition temperatures (*T*_g_) determined by DSC and DMA. The addition of ADP resulted in progressive embrittlement of HIPS with increasing concentration, while flammability decreased significantly and reached V-1 classification at loading of 30 wt.%. UV ageing caused photo-oxidative degradation of HIPS, which resulted in decreased strain-at-break, while flammability was not affected.

## 1. Introduction

Healthcare facilities (HSFs) are the main producers of healthcare waste. A term that is usually used to describe waste that is generated by HCFs is healthcare waste (HCW), but some other terms include medical waste, biomedical waste, clinical waste, and health facility waste [1,2]. Since the start of the SARS-CoV-2 virus pandemic, a large amount of medical waste has been generated. Medical waste is defined as a special pollutant with infectious, polluting, and toxic characteristics which is generated by HSFs in the process of medical diagnosis and treatment. Compared with general solid waste, medical waste has a higher risk of environmental pollution, because it usually carries many viruses, germs, chemical pollutants, and even radioactive materials. This presents a major challenge in the treatment procedures for medical waste, and appropriate measures should be taken to avoid this risk, depending on the exact content of the waste, as well as ensuring compliance with national regulations [2,3]. In recent decades, the production of medical waste has grown rapidly, with continuous advancements in medical technology and a remarkable increase in medical treatment [1]. In the European Union, the European Commission (EC) sets directives for waste regulations, and each nation is responsible for legislation that serves to fulfil these EC directives [4]. Biomarker analysis is one of the fundamental aspects of medical evaluation, and point-of-care (POC) tests have become valuable tools for rapid and sensitive analysis outside of the laboratory. One of the most used and important biosensors for POC diagnosis is lateral flow immunoassay (LFIA). LFIA is used for the qualitative detection of antibodies; it is performed outside of laboratories and provides results in a few minutes [5]. It works on the principle of immuno-chromatographic testing, based on the lateral traction of a liquid medium [6]. After the start of the coronavirus disease pandemic in December 2019 (COVID-19), mass rapid-antigen testing caused an enormous amount of medical waste to be produced. Rapid antigen tests (RATs) usually end up in an incineration process, which leads to new carbon dioxide (CO_2_) emissions. In addition, incineration results in the destruction of valuable raw materials, which could potentially be used in new applications. It is estimated that the incineration of one million RATs ends up destroying 1 g of gold nanoparticles (AuNPs) and 5000 kg of plastics [6]. Typically, RATs are composed of several porous pads–sample pad, conjugate pad, filter pads, nitrocellulose chromatographic membrane, and absorbent pad. Typically, the most used nanoparticles in conjugate pads are AuNPs. Test strips are usually put in a polymer cassette which provides good contact between various pads and membranes and protects them. Cassettes are typically composed of polystyrene (PS), high impact polystyrene (HIPS), and acrylonitrile butadiene styrene (ABS) plastics. Recycling of RATs involves the extraction of valuable AuNPs from the conjugate pads and plastic recycling, contributing to sustainability and resource optimization [7,8]. HIPS is a two-phase thermoplastic copolymer, composed of a continuous PS phase and a rubber phase [9]. It is prepared by graft copolymerization of PS and polybutadiene (PB) and contains 5–8% of rubber components [10]. That combination provides certain properties, such as excellent dimensional stability, rigidity, and good impact strength, which are necessary for the needs of many different applications. The disadvantages of HIPS include poor thermal stability, poor oxygen barrier properties, low ultraviolet (UV) light stability, and lower chemical resistance in comparison to semi-crystalline polymers. Because of its low cost and easy processing, HIPS is used in a large variety of industries and applications. The main industries include packaging and disposables, appliances, electronics and the electrical industry, toys and recreation, building products, and furnishings [9,11]. In many applications, in which products are exposed to potential ignition sources, such as in electrical and electronic equipment (EEE) the plastics flammability behaviour is of paramount importance. HIPS is very flammable and can flow and drip during burning, resulting in a faster spreading of the fire. Preventing the dripping effect during burning is important to prevent additional spreading of the fire. Therefore, plastics are often compounded with flame retardants to improve their burning behaviour. The halogenated flame retardants are the most used flame retardants for HIPS, which is a consequence of their low cost and high flame-retardant efficiency. Currently used halogenated flame retards for HIPS are: decabromodiphenyl oxide (DBDPO), ethylenebis(tetrabromophtalimide) (BT-93), octabromodiphenyl oxide (DE-79), 1,2-bis(pentabromophenyl), 2,4,6-tris(2,4,6-tribromophenoxy)-1,3,5-triazine, hexabromocyclododecane (HBCD), tetrabromobisphenol A, tetrabromobisphenol A bis (2,3-dibromopropyl ether), and 1,2-ethylenebis(tetrabromophthalimide) [12]. Thes fires caused by HIPS with halogenated flame retardants often result in the emission of toxic and harmful gases. Therefore, there is increasing interest in developing new halogen-free flame retardants, which present less risk to human health and the environment. The main groups of halogen-free flame retardants include metal hydroxides, intumescent flame retardants, nanoparticles, and flame retardants containing different flame-retardant elements (e.g., nitrogen, phosphorus, and silicon) [10,13]. Phosphorous-based flame retardants cover a wide range of inorganic and organic compounds, which can be both reactive products and additives. Reactive products are chemically bonded in polymers, while additives are integrated into the material during mixing. Phosphorus-based flame retardants have a broad range of applications and good fire retardancy properties. The most used phosphorus-based flame retardants are phosphate esters, phosphonates, and phosphinates, and more specifically, triphenyl phosphate (TPP), resorcinol bis (diphenylphosphate) (RDP), Bisphenol A diphenylphosphate (BADP), tricrecyl phosphate (TCP), and ADP. ADP can be classified in the group of organic phosphinates and has an excellent flame-retardant effect, as it works both in the condensed phase (formation of carbon) and the combustion zone (removal of high-energy free radicals). Another benefit of ADP is that it usually has only a small effect on the mechanical properties. Therefore, it is an excellent candidate for the preparation of flame-retardant plastics. However, there are some challenges in the application of ADP, such as its poor compatibility with the polymer matrix, which can significantly affect flame retardancy and mechanical properties [14,15,16]. A recent study by ToxServices LLC evaluated the toxicity and environmental concerns of ADP and assigned it a GreenScreen Benchmark^TM^ score of 3, signifying low levels of concern for most endpoints and some non-critical data gaps [17]. Despite its attractive flame-retardant properties and low health and environmental hazards, there are no studies dealing with its effects on HIPS, according to our best knowledge.

The present study aimed to explore the potential of waste RATCs as a secondary raw material for high-value-added EEE applications. Milled waste RATCs were compounded with varying concentrations of non-halogen flame retardant ADP to obtain a sustainable plastic with low flammability. As HIPS is known to have poor UV stability, which is especially critical when using secondary raw materials and can also affect the efficiency of flame retardants, the samples were exposed to UV ageing. The underlying photodegradation mechanisms were studied by means of the changes in the chemical composition using FT-IR spectroscopy, and the corresponding changes in mechanical and thermal properties as well as burning behaviour were studied.

## 2. Materials and Methods

### 2.1. Materials

Milled SARS-CoV-2 RATCs were received from a waste management company (Surovina d.o.o., Maribor, Slovenia). The material was milled below a size of 4 mm. The flame retardant used in the study was Omnistab FR ADP (Partners in Chemicals, Utrecht, The Netherlands), which is chemically an ADP.

### 2.2. Sample Preparation

#### 2.2.1. Compounding

Milled test cassettes were compounded using LTE 20-44 (Labtech Engineering, Samut Prakan, Thailand) co-rotating twin-screw extruder. The extruder had a screw diameter of 20 mm and an L:D ratio of 44:1. Milled material was fed into the main funnel with a single screw loss-in-weight feeding unit, MCBalance (Movacolor, Wolkammersstraat, The Netherlands). Flame retardant was fed downstream using a side feeding system equipped with a twin-screw loss-in-weight feeding unit, MCPowder (Movacolor, Wolkammersstraat, The Netherlands). The total mass flow of materials was 10 kg/h and the screw angular velocity was 200 min^−1^. Table 1 presents the temperature profile of the extrusion.

#### 2.2.2. Injection Moulding

The test specimens were prepared using a CX 50-180 (Krauss Maffei, München, Germany) injection moulding machine. The machine had a clamping force of 500 kN, an L/D ratio of 23.3, and a screw diameter of 30 mm. The produced parts consisted of a specimen for tensile tests produced according to the ISO 527-2 standard (type 1BA) and specimens for impact testing (notched and unnotched) manufactured according to the ISO 179 standard [18,19]. The material was dried before injection moulding in a DryWell (Vismec Srl, Camposampiero, Italy) dryer at 80 °C, below a moisture content of 0.1%. The injection moulding processing parameters are summarised in Table 2.

#### 2.2.3. UV Aging

Samples were irradiated with an IntelliRay 600 (Uvitron, West Springfield, MA, USA) UV flood curing system equipped with a 600 W UVA metal halide lamp at 35% intensity. Samples were irradiated for various durations (2 h interval, up to 10 h) and were flipped after half the time of the total irradiation.

### 2.3. Characterisation

#### 2.3.1. Fourier-Transform Infrared Spectroscopy

FT-IR spectra were recorded on a Perkin Elmer Spectrum 65 (Perkin Elmer, Waltham, MA, USA) FT-IR spectrometer using an attenuated total reflection (ATR) technique with a spectral resolution of 4 cm^−1^ and a measuring range from 4000 cm^−1^ to 600 cm^−1^. Obtained spectra were averaged over 16 scans.

#### 2.3.2. Differential Scanning Calorimetry

Thermal properties were determined using a DSC 2 (Mettler Toledo, Greifensee, Switzerland) calorimeter and 40 µL aluminium crucibles. Specimens were prepared from tensile test specimens and had a mass of 5.0–15.0 mg. The samples were tested in the temperature range of −80 °C to 180 °C, with a heating rate of 10 K/min in the N_2_ atmosphere (20 mL/min). The isothermal segments before the heating and cooling segments were set to 1 min.

#### 2.3.3. Thermogravimetric Analysis

The decomposition behaviour was investigated using a TGA/DSC 3+ (Mettler Toledo, Greifensee, Switzerland) simultaneous TGA/DSC instrument in 40 µL aluminium crucibles. Isothermal measurements were performed by heating the samples from 40 °C to 550 °C with a heating rate of 10 K/min in the nitrogen (N_2_) atmosphere (20 mL/min), followed by a 10 min isothermal segment in oxygen (O_2_) atmosphere (20 mL/min).

#### 2.3.4. Dynamic Mechanical Analysis

Dynamic mechanical properties were determined using a DMA 8000 (Perkin Elmer. Waltham, MA, USA) dynamic mechanical analyser according to the ASTM D5418 standard [20]. Specimens were prepared from tensile test specimens. Samples were tested in flexure using a dual cantilever beam support. The distance between the supports was 12.6 mm, the frequency was 1 Hz, and the amplitude was 0.02 mm. Samples were tested from 28 °C to 130 °C, with a heating rate of 2 K/min.

#### 2.3.5. Tensile Tests

Tensile properties were determined using an Ag-X plus 10 kN (Shimadzu, Kyoto, Japan) universal testing machine according to the ISO 527-1 standard [21]. The gauge length was 50 mm, the preload was 10 N, and the testing speed was 1 mm/min until 0.25% strain and 50 mm/min until the break. The results are average values of five measurements.

#### 2.3.6. Impact Strength Measurements (Charpy)

Charpy impact properties were determined using an LY-XJJDS (LIYI, Dongguan, China) pendulum impact tester according to ISO 179, with a 1 J pendulum (notched samples). The distance between supports (span) was 60 mm. The tests were carried out at room temperature. The results are an average of 10 measurements.

#### 2.3.7. Vertical Burning Test

The vertical burning behaviour was evaluated according to the D3801 standard test method for measuring the comparative burning characteristics of solid plastics in a vertical position. This test (or method) and the 200 mm (50 W) vertical burning test (V0, V-1, or V-2) of ANSI/UL 94 are equivalent. The procedure consists of subjecting a set of preconditioned specimens of identical composition and geometry to a standard flame for two 10 s flame applications. After the first flame application, afterflame time is measured (*t*_1_), and after the second flame application, afterflame (*t*_2_) and afterglow times are recorded. During the test it is also observed whether material drips from the material and whether these drips ignite a cotton indicator. Important information is also the total flame time for each specimen set. Tested samples had dimensions of 80 × 10 × 40 mm [22].

## 3. Results

### 3.1. Fourier-Transform Infrared Spectroscopy

**Figure 1:**
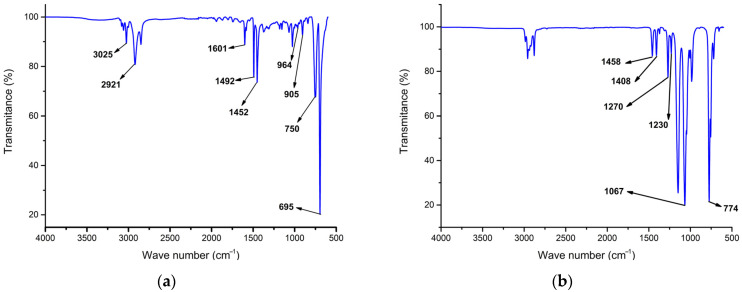
FT-IR spectra of (**a**) unmodified recycled material from RATCs and (**b**) flame retardant (ADP).

The chemical composition of prepared recyclates was identified by recording FT-IR spectra and performing a pattern matching by using a spectral database, which revealed that RATCs are composed mainly of HIPS. Figure 1a presents a recorded spectrum of unmodified recycled material from RATCs, with marked characteristic absorption bands for HIPS. The analysed sample exhibited absorption bands attributed to C-H stretching vibrations in the region between 3200–2800 cm^−1^, with aromatic C-H vibrations at 3086 cm^−1^, 3063 cm^−1^, and 3026 cm^−1^, and asymmetrical and symmetrical stretching vibrations of the methylene -CH_2_ groups at 2922 cm^−1^ and 2850 cm^−1^. Absorption bands attributed to C-C stretching of the benzene ring were found at 1602 cm^−1^, 1490 cm^−1^, and 1446 cm^−1^; C-C stretching and C-H bending vibrations were found at 1185 cm^−1^ and 1157 cm^−1^, in-plane C-H bending vibrations of the aromatic ring at 1066 cm^−1^ and 1028 cm^−1^, and out of-plane C-H bending vibrations between 900 cm^−1^ and 675 cm^−1^. The two bands with the highest transmittance, at 752 cm^−1^ and 693 cm^−1^, were attributed to the vibrations of monosubstituted benzene rings. The band around 964 cm^−1^ corresponds to the C=C vibration of trans-1,4-double bonds present in polybutadiene (PB) [23,24]. An FT-IR spectrum of the flame retardant is shown in Figure 1b. Absorption bands at 1463 cm^−1^ and 1381 cm^−1^ were assigned to bending vibrations of C-H and -CH_3_. Further, the absorption bands at 1463 cm^−1^ and 1381 cm^−1^ were related to the vibrations of P=O and P-C bonds. A band at 780 cm^−1^ was assigned to the stretching vibration of P=C. The next bands were strong and sharp bands at 2939 cm^−1^, 2900 cm^−1^, and 2833 cm^−1^, which represented the stretching vibration of CH_3_(CH_2_)- groups [25,26].

**Figure 2:**
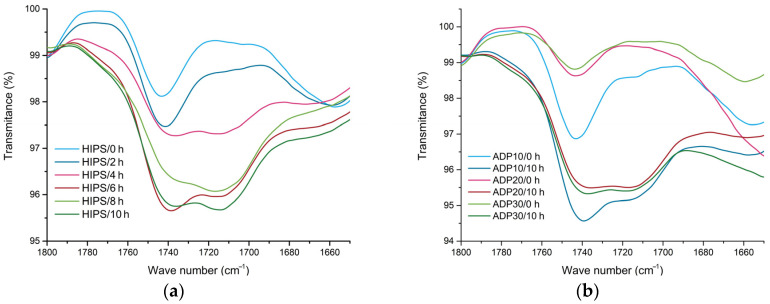
Comparison of FT-IR absorption bands attributed to carbonyl groups of (**a**) recycled HIPS exposed to UV ageing for various durations and (**b**) recycled HIPS containing different concentrations of ADP at different time steps of UV ageing for recycled material.

In Figure 2, a comparison of the carbonyl region in the FT-IR spectrum is made for the different time steps of UV ageing. Samples that were not exposed to UV ageing exhibited an absorption band at 1740 cm^−1^ ascribed to the carbonyl group, containing additives such as processing aids or other unknown impurities. With increased time of UV ageing an increase of the transmittance of the absorption band at 1740 cm^−1^ was observed as well as the formation of an additional band at about 1720 cm^−1^, ascribed to the ester/aldehyde groups and carboxylic acid groups, respectively, and resulting from photooxidation reactions. The changes in carbonyl group absorption bands related to the ester/aldehyde groups (1720 cm^−1^) were slightly less pronounced upon UV exposure for samples containing ADP, which may be due to decreased UV penetration into the matrix. Polybutadiene is the main oxidation point of HIPS. The inclusion of PB is beneficial for toughening, but it provides a fragile site for the initiation of photo-oxidative degradation. UV degradation mainly occurs at wavelengths ≥300 nm with the formation of radicals at the allylic position in PB. When oxygen is present formation of unstable alkyl and peroxyl radicals and photo-unstable intermediary products occurs. These products produce further radicals with UV irradiation, resulting in cross-linking and chain scission together with the formation of lower molecular weight products [25,27,28,29,30,31].

### 3.2. Differential Scanning Calorimetry (DSC)

**Figure 3:**
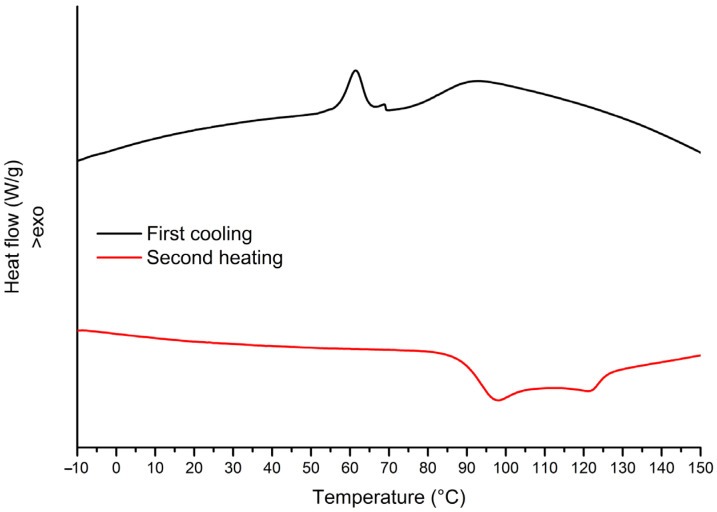
DSC thermograms (first cooling and second heating run) of unmodified materials from RATCs.

In Figure 3, the DSC thermograms of the unmodified recycled material from RATCs are presented. Upon cooling, a change of specific heat capacity (Δ*C*_p_) corresponding to the glass transition can be observed at 89.4 °C, in addition to an exothermic peak at 61.4 °C, which was attributed to the crystallization of an unknown compound (processing aids or impurities) [32]. Upon heating, a Δ*C*_p_ due to glass transition was observed at 91.6 °C, followed by a small endothermic relaxation peak and an endothermic peak at 121.0 °C ascribed to the melting of unknown additives.

Table 3 summarizes the thermal properties of samples determined by DSC. Reported *T*_g_ and Δ*C*_p_ were determined at the second heating run. The measured *T*_g_ of samples ranged from 89 °C to 92 °C, without clear trends of the UV exposure or ADP content on the *T*_g_. The only significant difference in thermal properties determined by DSC was found for Δ*C*_p_, which decreased with increasing ADP content due to there being less polymer in the sample.

### 3.3. Thermogravimetric Analysis

**Figure 4:**
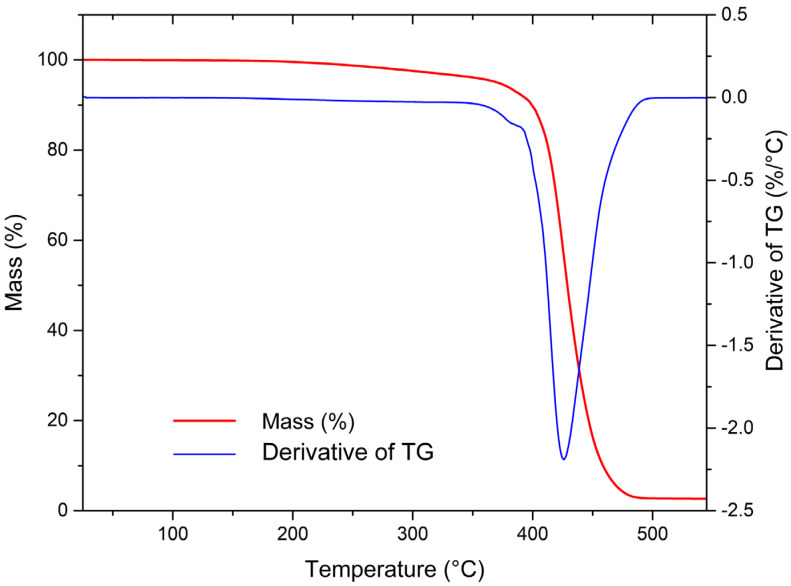
TGA and DTG thermograms of unmodified recycled HIPS from RATCs.

Representative TGA and derivative thermogravimetry (DTG) thermograms of unmodified recycled HIPS are shown in Figure 4. The main decomposition occurred in a single step, with a decomposition temperature (*T*_d_) of 428.5 °C and a mass loss step (ΔY_1_) of 97.4%. After switching to an oxygen atmosphere an additional mass loss of 0.4% was observed, which was attributed to the decomposition of char, while the total residue equated to 2.2% and was attributed to the inorganic fillers. Similar TGA and DTG curves were observed for all samples, with corresponding parameters presented in Table 4. The largest differences in the decomposition behaviour were observed with the addition of ADP, which resulted in increased *T*_d_ of 4.8 °C and 4.4 °C at concentrations of 20 wt.% and 30 wt.%, respectively. Slight variations in char and ash content between the samples may also be attributed to the inhomogeneity of the input material.

### 3.4. Dynamic Mechanical Analysis

**Figure 5:**
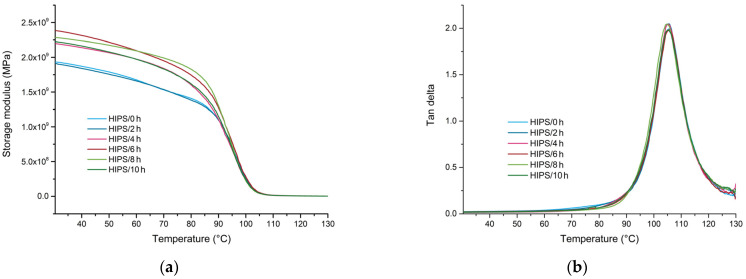
DMA results of recycled HIPS exposed to various durations of UV: (**a**) comparison of storage modulus as a function of temperature; and (**b**) comparison of tan delta as a function of temperature.

Figure 5 presents DMA thermograms of unmodified recycled HIPS exposed for various durations, while corresponding values of storage modulus determined at 30 °C and 100 °C and loss factor (tan delta) peak temperatures (*T*_g_) are presented in Table 5. Storage modulus below the *T*_g_ increased significantly upon UV exposure of more than 4 h. Storage modulus at 30 °C increased from around 1940 MPa to 2200 MPa for a UV exposure time of 4 h and further increased to around 2300 MPa after 6 h of UV exposure. With further increases of exposure time to 8 h and 10 h, a slight decrease in storage modulus to approximately 2290 MPa and 2230 MPa was observed, respectively. There are two possible mechanisms for the increase of storage modulus upon UV ageing. First, an increase in storage modulus could be a consequence of the photooxidation reactions of double bonds in polybutadiene and cross-linking of polymer chains, which results in a more rigid rubbery phase. In addition, the increased storage modulus could also be a consequence of the decrease in free volume. Physical ageing of the PS phase causes structural recovery of the polymeric chains towards lower energy states; this results in a decrease of the free volume and, in turn, higher cohesion of polymeric chains, lower molecular mobility and consequently increased storage modulus [9]. In contrast, no significant differences in tan delta peak temperatures were observed for samples UV-aged for different durations.

**Figure 6:**
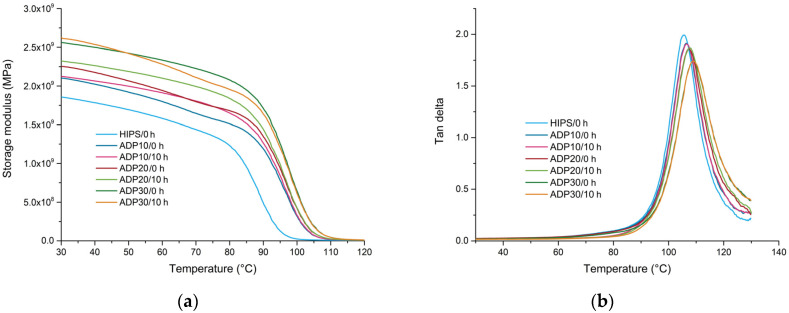
DMA results for recycled HIPS containing various concentrations of ADP (untreated and UV-aged for 10 h): (**a**) storage modulus as a function of temperature; and (**b**) tan delta as a function of temperature.

In Figure 6, DMA thermograms of samples containing various concentrations of ADP (untreated and UV-aged for 10 h) are presented, with the corresponding values listed in Table 5. Increasing the concentration of ADP resulted in increased storage modulus over the whole examined temperature range and increased glass transition temperatures. The increase of storage modulus effect is especially prominent at temperatures in the region of glass transition, where an increase in the storage modulus of more than 2.5-fold was observed at 100 °C. The results indicate that ADP acts as a particulate filler, with relatively good interactions with the HIPS matrix as indicated by the increased glass transition temperatures. The effect of UV ageing on the properties of ADP-modified HIPS was not as distinct as for unmodified polymers. A slight increase of storage modulus was observed for UV-aged samples containing 10 wt.% and 20 wt.% of ADP, while storage modulus at 30 °C of samples containing 30 wt.% even slightly decreased. Samples containing ADP are significantly stiffer in comparison to the neat matrix, thus an increased storage modulus of the matrix may not have such a distinct contribution to the modulus of the composite. In addition, the ADP may prevent the penetration of UV light into the matrix, preventing the decomposition reactions, a determination which is also suggested by FT-IR spectroscopy.

### 3.5. Tensile Tests

**Figure 7:**
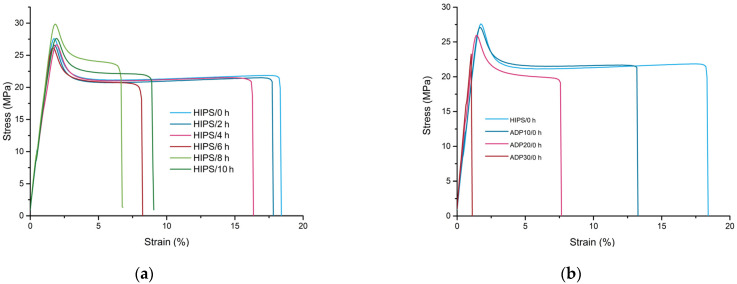
Comparison of representative stress–strain diagrams: (**a**) recycled HIPS exposed to various durations of UV ageing; and (**b**) recycled HIPS containing various concentrations of ADP (untreated and UV-aged for 10 h).

In Figure 7a stress–strain curves for samples of recycled and UV-aged samples with different time steps are presented, with corresponding mechanical properties listed in Table 6. The main changes in the mechanical properties of HIPS upon UV exposure were observed for strain-at-break, which decreased from around 20% to a range of 7–9% for samples UV-aged between 6 h to 10 h. Cross-linking increases the rigidity of the PB domains and eliminates their toughening effectiveness, which results in the crack propagation seen along the interface between the PB domains and the PS. The resulting reduction in impact performance is accompanied by dramatic reductions in elongation at break, with these properties approaching those of pure PS [31]. It seems that loss-in-flexibility plateaued at a UV exposure time of around 6 h to 10 h. This effect may be related to the saturation of the crosslinking density of the PB phase, where, usually, the first main chemical changes occur. However, it can be expected that upon additional UV exposure, additional loss of mechanical properties would occur due to chain scission of the PS phase [31]. In Figure 7b stress–strain diagrams of recycled HIPS containing various concentrations of ADP are presented. The addition of ADP resulted in increased tensile modulus and decreased strain-at-break, while tensile strength was not influenced significantly. Tensile modulus increased from about 1.9 GPa to about 3.5 GPa, while strain-at-break decreased from about 20% to only about 1% for samples containing 30 wt.% of ADP. This significant embrittlement of HIPS at high concentrations of ADP may be a limiting factor for some applications for which high levels of toughness are required. Upon UV exposure of ADP-modified samples, an additional decrease in strain-at-break occurred for samples containing 10 wt.% and 20 wt.% of ADP. In contrast, strain-at-break was unchanged after UV exposure for a sample containing 30 wt.% of ADP. This phenomenon may be attributed to an already very low strain at the break of the composites. As a result, crack formation and propagation occur mainly at the matrix–filler interphase, while embrittlement of the matrix due to degradation reactions doesn’t contribute to premature failure.

### 3.6. Charpy Impact Strength

**Figure 8:**
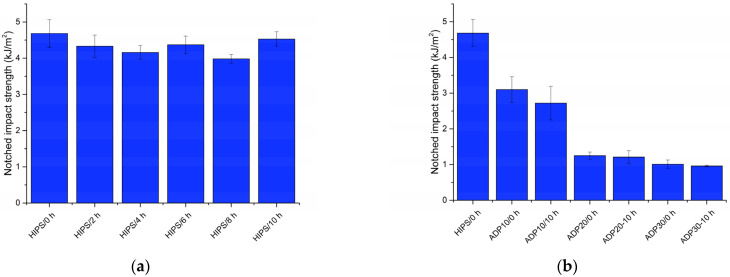
Results of notched Charpy impact tests: (**a**) recycled HIPS exposed to various durations of UV ageing; and (**b**) recycled HIPS containing various concentrations of ADP (untreated and UV-aged for 10 h).

Figure 8a presents the Charpy impact strengths of unmodified HIPS recyclates which were UV-aged for various durations. The Charpy impact strength ranged from around 4.0 kJ/m^2^ to 4.5 kJ/m^2^, and was independent of the UV ageing time. The Charpy impact test results are in contrast to the tensile test results, in which UV ageing resulted in significant embrittlement of the HIPS. This may be partially related to the higher thickness of Charpy specimens (4 mm instead of 2 mm) and, in turn, the lower degradation in the centres of the specimens. The results in Figure 8b present the notched Charpy impact strengths of recycled HIPS containing various concentrations of ADP (untreated and UV-aged for 10 h). The impact strength decreased from about 4.7 kJ/m^2^ to about 3.0 kJ/m^2^ at 10 wt.% of ADP. Increase in the ADP content to 20 wt.% resulted in a significant decrease in impact strength to about 1.3 kJ/m^2^, while a further increase of ADP loading to 30 wt.% resulted in only a slight decrease of impact strength to about 1.0 kJ/m^2^. Similarly, as for the unmodified recycled HIPS, the UV ageing didn’t significantly influence the impact strength of HIPS containing ADP.

### 3.7. Vertical Burning Tests

The results of the vertical burning test and a classification for each material are presented in Table 7. The vertical burning test classifies solid plastics according to their burning behaviour into different classes (V-2, V-1, and V-0). For V-2 classification material, afterflame time for each specimen (*t*_1_ or *t*_2_) should be lower than 30 s, total afterflame (*t*_1_ or *t*_2_) for a set of five specimens should be lower than 250 s, and afterflame plus afterglow time after the second flame application should be lower than 60 s, while the cotton indicator below the sample could be ignited by flaming particles or drops. For the V-1 classification all requirements associated with afterflame times are the same as for the V-2 classification; the only difference is that the material should not exhibit dripping behaviour and there should be no ignition of the cotton indicator with flaming drops or particles. A sample containing 10 wt.% of ADP couldn’t be classified in any of the flammability ratings. A sample containing 20 wt.% of ADP could be classified with a V-2 rating. It failed to be classified in the V-1 rating because it exhibited dripping behaviour during burning. While the average values of *t*_1_ and *t*_2_ were below the requirements of the V-0 rating, the sample failed to reach them due to a few outliers which felt out of the required range. UV exposure resulted in increased average values of both afterflame times (*t*_1_ and *t*_2_) for samples containing 20 wt.% and 30 wt.% of ADP, however, the differences were not statistically significant, due to high variance.

## 4. Conclusions

The present study aimed to evaluate the potential of waste RATCs as secondary raw materials for the preparation of sustainable flame-retardant plastics which can be used in the EEE. Milled RATCs were compounded with different concentrations (10–30 wt.%) of ADP, injection moulded into test specimens, and characterised. FT-IR spectroscopy revealed that RATCs are predominately made from HIPS. Increasing duration of UV ageing resulted in the formation of additional carbonyl groups, which were attributed to photooxidation reactions occurring mainly at the PB phase. According to the DSC analysis, samples exhibited glass transitions ranging from 89 °C to 92 °C, independent of the composition, while the presence of additional melting and crystallisation peaks indicates the impurities or processing aids. The addition of 20 wt.% and 30 wt.% of ADP resulted in increased decomposition temperatures for 4.8 °C and 4.4 °C, respectively. UV ageing resulted in increased storage modulus, which was attributed to the crosslinking reaction between double bonds of the PB phase and/or a decrease in free volume. With increasing ADP concentration, the storage modulus of HIPS increased, especially in the glass transition region, indicating that ADP acts as a particulate filler with relatively good interactions with the matrix. UV exposure resulted in a loss in strain-at-break for HIPS, while notch Charpy impact strength was not affected significantly. The addition of ADP to HIPS resulted in higher tensile modulus, but progressive embrittlement was evident in losses in strain-at-break and Charpy impact strength, which was especially critical at 30 wt.% loading. Samples containing 20 wt.% and 30 wt.% of ADP reached V-2 and V-1 flammability classifications, respectively. A sample with 30 wt.% almost reached the V-0 classification, with only a few outliers being outside of the required specifications. With further fine-tuning of processing parameters or material composition, achieving V-0 flammability classification may be possible. Overall, the results indicate the high potential of waste RATCs as a secondary raw material for new applications, such as in EEE. By compounding them with ADP, a flame-retardant plastic for EEE could be obtained with potentially lower environmental impacts and health hazards compared to currently used primary materials. However, further development and testing may be needed to ensure compliance with other relevant requirements, such as electric properties. Moreover, proper management of the environmental and health hazards due to the potentially infectious character of the waste should be established to enable the recycling of RATCs on an industrial scale.

## Figures and Tables

**Table 1:** The temperature profile of the compounding.

Zone	Die	10	9	8	7	6	5	4	3	2	1
T (°C)	190	190	185	185	180	180	175	175	170	160	140

**Table 2:** Processing parameters.

Parameter	Value
Barrel temperature-hopper to nozzle	200 °C, 210 °C, 200 °C, 190 °C, 180 °C
Injection velocity profile	60 mm/s, 20 mm/s last 2 mm
Switchover point	11 mm
Packing pressure profile	40 MPa (10 s), 10 MPa (1 s)
Metering stroke	20 mm
Screw angular velocity	100 min^−1^
Backpressure	10 MPa
Cooling time	10
Mould temperature	40 °C
Rest cooling time	10 s

**Table 3:** Thermal properties of samples determined by DSC.

Sample	*T*_g_ (°C)	Δ*C*_p_ (J/gK)	*T*_c_ (°C)	Δ*H*_c_ (J/g)	*T*_m1_ (°C)	Δ*H*_m1_ (J/g)
HIPS/0 h	91.6	0.19	61.4	0.7	121.0	0.6
HIPS/2 h	89.1	0.19	62.8	0.7	119.7	0.7
HIPS/4 h	89.8	0.18	62.7	0.7	119.3	0.6
HIPS/6 h	91.7	0.16	60.5	0.7	122.1	0.5
HIPS/8 h	90.7	0.20	62.5	0.7	120.0	0.6
HIPS/10 h	89.0	0.18	62.3	0.8	119.6	0.6
ADP10/0 h	90.8	0.16	61.4	0.3	120.9	0.3
ADP10/10 h	90.0	0.16	62.6	0.2	120.4	0.3
ADP20/0 h	91.3	0.15	60.2	0.1	120.8	0.4
ADP20/10 h	91.8	0.14	61.6	0.0	120.4	0.2
ADP30/0 h	91.3	0.13	61.8	0.0	120.0	0.2
ADP30/10 h	90.9	0.12	61.7	0.0	119.7	0.2

**Table 4:** Thermal properties determined by TGA.

Sample	*T*_d_ (°C)	ΔY_1_ (%)	Char (%)	Ash (%)
HIPS/0 h	428.5	97.4	0.4	2.2
HIPS/2 h	428.8	97.2	0.4	2.4
HIPS/4 h	428.5	97.3	0.4	2.3
HIPS/6 h	428.6	98.2	0.4	1.4
HIPS/8 h	429.2	98.0	0.4	1.6
HIPS/10 h	429.2	97.7	0.2	2.1
ADP10/0 h	428.4	98.2	0.7	1.1
ADP10/10 h	428.0	97.3	0.9	1.8
ADP20/0 h	433.3	97.7	0.5	1.8
ADP20/10 h	430.1	96.7	0.8	2.5
ADP30/0 h	432.9	98.2	0.3	1.5
ADP30/10 h	433.7	97.8	0.4	1.8

**Table 5:** Values of storage modulus at 30 °C and 100 °C and tan delta peak temperatures (*T*_g_) of samples.

Sample	Storage Modulus at 30 °C (MPa)	Storage Modulus at 100 °C (MPa)	Tan Delta Peak (°C)
HIPS/0 h	1942.4	232.0	105.4
HIPS/2 h	1912.3	222.4	105.1
HIPS/4 h	2200.0	196.3	105.3
HIPS/6 h	2388.7	243.1	105.6
HIPS/8 h	2287.1	185.4	104.8
HIPS/10 h	2229.4	202.4	105.2
ADP10/0 h	2106.3	290.5	106.3
ADP10/10 h	2125.8	307.1	106.6
ADP20/0 h	2261.1	395.9	107.4
ADP20/10 h	2323.4	404.9	107.6
ADP30/0 h	2626.5	592.1	108.9
ADP30/10 h	2560.6	622.2	109.1

**Table 6:** Mechanical properties determined by tensile tests.

Sample	Tensile Modulus (GPa)	Tensile Strength (MPa)	Strain-sat-Break (%)
HIPS/0 h	1.87 ± 0.08	26.8 ± 0.6	20.1 ± 3.8
HIPS/2 h	2.08 ± 0.10	26.8 ± 0.2	16.4 ± 3.7
HIPS/4 h	2.06 ± 0.13	26.6 ± 0.2	16.7 ± 0.9
HIPS/6 h	1.91 ± 0.17	26.2 ± 0.2	7.7 ± 0.5
HIPS/8 h	1.97 ± 0.06	28.4 ± 1.5	7.0 ± 0.3
HIPS/10 h	1.74 ± 0.19	27.2 ± 0.8	8.6 ± 0.3
ADP10/0 h	2.61 ± 0.61	26.0 ± 1.3	12.9 ± 2.0
ADP10/10 h	2.37 ± 0.22	27.0 ± 0.2	4.8 ± 1.7
ADP20/0 h	3.03 ± 0.18	26.0 ± 0.2	6.1 ± 1.1
ADP20/10 h	2.28 ± 0.07	27.0 ± 0.1	1.9 ± 0.2
ADP30/0 h	3.48 ± 0.35	25.3 ± 1.1	1.2 ± 0.1
ADP30/10 h	3.06 ± 0.47	25.3 ± 0.9	1.1 ± 0.1

**Table 7:** Vertical burning test results.

Sample	*t*_1_ (s)	*t*_2_ (s)	Burning Droplets	Classification
ADP10/0 h	66.8 ± 9.8	/	Yes	/
ADP10/10 h	42.0 ± 5.8	/	Yes	/
ADP20/0 h	6.0 ± 3.5	17.6 ± 16.7	Yes	V-2
ADP20/10 h	15.0 ± 8.1	20.8 ± 17.1	Yes	V-2
ADP30/0 h	3.5 ± 1.7	5.6 ± 2.4	No	V-1
ADP30/10 h	5.6 ± 2.4	15.3 ± 10.3	No	V-1

## Data Availability

Data are contained within the article.
